# Investigation of Copy Number Variation in South African Patients With Congenital Heart Defects

**DOI:** 10.1161/CIRCGEN.121.003510

**Published:** 2022-10-07

**Authors:** Nicole A. Saacks, James Eales, Timothy F. Spracklen, Thomas Aldersley, Paul Human, Mark Verryn, John Lawrenson, Blanche Cupido, George Comitis, Rik De Decker, Barend Fourie, Lenise Swanson, Alexia Joachim, Andre Brooks, Raj Ramesar, Gasnat Shaboodien, Bernard D. Keavney, Liesl J. Zühlke

**Affiliations:** 1 Division of Pediatric Cardiology, Department of Pediatrics and Child Health (N.A.S., T.F.S., T.A., J.L., G.C., R.D.D., L.S., A.J., L.J.Z.).; 2 Department of Medicine, Cape Heart Institute (T.F.S., G.S., L.J.Z.).; 3 Chris Barnard Division of Cardiothoracic Surgery, Department of Medicine, Faculty of Health Sciences (P.H., A.B.).; 4 Division of Cardiology, Department of Medicine, Groote Schuur Hospital, Faculty of Health Sciences (B.C., L.J.Z.).; 5 MRC Genomic & Precision Medicine Research Unit, Division of Human Genetics, Dept of Pathology, Institute for Infectious Diseases and Molecular Medicine, University of Cape Town, Cape Town, South Africa (R.R.).; 6 Division of Cardiovascular Sciences, School of Medical Sciences, Faculty of Biology, Medicine and Health, The University of Manchester, United Kingdom (J.E., B.D.K.).; 7 Cardiovascular Genetics Laboratory, Hatter Institute for Cardiovascular Research in Africa (M.V., G.S.).; 8 Division of Pediatric Cardiology, Department of Pediatrics and Child Health, University of Stellenbosch, Cape Town, South Africa (J.L., B.F.).; 9 Heart Institute, Manchester Univ NHS Foundation Trust, Manchester Academic Health Science Centre, United Kingdom (B.D.K.).; 10 South African Medical Research Council, Cape Town (L.J.Z.).

**Keywords:** chromosomal abberations, Africa, southern, DNA copy number variations, genetics, heart defects, congenital

## Abstract

Congenital heart disease (CHD) is a leading non-infectious cause of pediatric morbidity and mortality worldwide. Although the etiology of CHD is poorly understood, genetic factors including copy number variants (CNVs) contribute to the risk of CHD in individuals of European ancestry. The presence of rare CNVs in African CHD populations is unknown. This study aimed to identify pathogenic and likely pathogenic CNVs in South African patients with CHD.

**Methods:**

Genotyping was performed on 90 patients with nonsyndromic CHD using the Affymetrix CytoScan HD platform. These data were used to identify large, rare CNVs in known CHD-associated genes and candidate genes.

**Results:**

We identified eight CNVs overlapping known CHD-associated genes (*GATA4*, *CRKL*, *TBX1*, *FLT4*, *B3GAT3*, *NSD1*) in six patients. The analysis also revealed CNVs encompassing five candidate genes likely to play a role in the development of CHD (*DGCR8*, *KDM2A*, *JARID2*, *FSTL1*, *CYFIP1*) in five patients. One patient was found to have 47, XXY karyotype. We report a total discovery yield of 6.7%, with 5.6% of the cohort carrying pathogenic or likely pathogenic CNVs expected to cause the observed phenotypes.

**Conclusions:**

In this study, we show that chromosomal microarray is an effective technique for identifying CNVs in African patients diagnosed with CHD and have demonstrated results similar to previous CHD genetic studies in Europeans. Novel potential CHD genes were also identified, indicating the value of genetic studies of CHD in ancestrally diverse populations.

Congenital heart disease (CHD) is the most prevalent birth defect, affecting around 9 of every 1000 children born worldwide.^1^ An estimated 300 000 African children are born with this condition each year.^2^ Although CHD is a leading noninfectious cause of pediatric morbidity and mortality worldwide,^3^ knowledge of its causes remains relatively poor.

CHD has a diverse and incompletely understood etiology, comprising both environmental causes and genetic factors. A cause of disease may not be identified in more than half of the patients with CHD, although genetic mutations and chromosomal rearrangements have been demonstrated to play an important role in the pathogenesis of CHD.^4^ As a complex disorder, the genetic factors in CHD range from rare, deleterious mutations to more common susceptibility loci. Advanced genetic techniques including whole-exome sequencing and chromosomal microarray have revealed numerous rare single nucleotide variants and copy number variants (CNVs) associated with CHD.^5–9^ The discovery of disease-causing CNVs associated with CHD has improved our understanding of the etiology of the disease,^4^ with the identification of dosage-sensitive genes that are critical for cardiac development. An increased CNV burden of 1.8- to 3.9-fold has been reported in CHD cases compared with controls, with large, rare, gene-containing CNVs having a greater impact on CHD.^7,10^ Up to 10% of CHD is thought to be due to the effects of rare CNVs in the chiefly European-ancestry populations that have been studied to date.

The genetic basis of CHD in Africa has been minimally explored. A recent study of CHD in Nigeria reported a mutation rate of 9% using exome sequencing, as well as an undisclosed number of disease-causing CNVs in the patient population.^11^ Because of the vast genetic diversity of African populations, similar improvements in knowledge of the genetic architecture of CHD elsewhere in Africa will help define disease risk in this population, advance the management and treatment of patients, and facilitate prevention. The PROTEA (Partnerships for Congenital Heart Disease in Africa) Study was established to investigate the epidemiology and genetics of CHD in sub-Saharan Africa and incorporated a densely phenotyped registry of African patients with CHD.^12^ Initial analysis of the PROTEA cohort has indicated lower proportions of mild and moderate CHD subtypes, likely due to underdiagnosis in the Western Cape Province of South Africa, similar to other regions in Africa.^12^ The aim of this PROTEA study subset was to determine the prevalence of disease-causing CNVs in a South African CHD cohort.

## Methods

Full methods are available in the Supplemental Material. The data that support the findings of this study are available from the corresponding author upon reasonable request. The filtered CNV calls have been made publicly available (10.25375/uct.11953749). Data from consortia were accessed subject to the applicable data-sharing agreements. The Human Research Ethics Committee of the University of Cape Town approved all procedures (reference no. 389/2019), and written informed consent was obtained from all participants.

## Results

### Study Cohort

Chromosomal microarray was conducted on 90 patients presenting with an isolated heart defect (n=72) or CHD with additional extracardiac anomalies (ECAs; n=18). The study cohort consisted of 50 females (55.6%) and 40 males (44.4%) and was predominantly of the Cape mixed ancestry population (65.6%; Table 1). The study patients presented with a wide spectrum of cardiac phenotypes (Table 2). The four most frequent CHD diagnoses were Tetralogy of Fallot (TOF; n=13), isolated ventricular septal defect (n=11), patent ductus arteriosus (n=10), and pulmonary atresia (n=8).

**Table 1.**
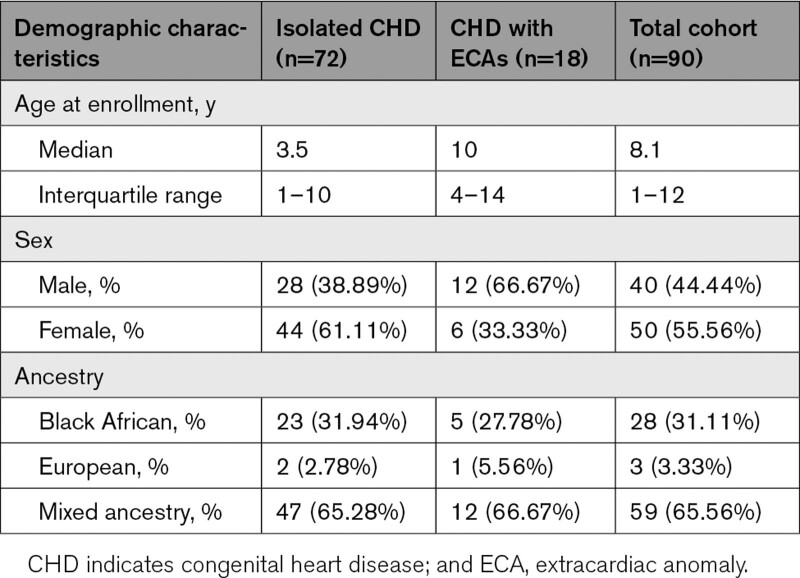
Demographic Characteristics of the Study Cohort

**Table 2.**
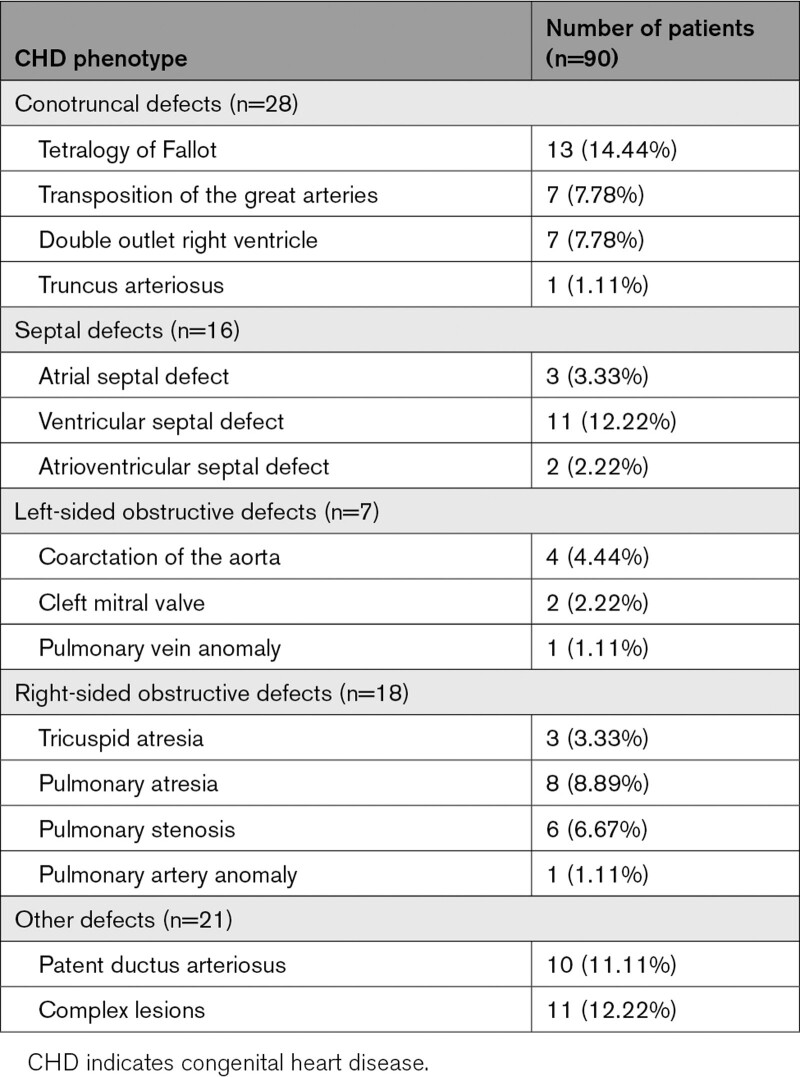
Phenotypic Characteristics of the Study Cohort

### Identification of Pathogenic CNVs

**Figure 1.**
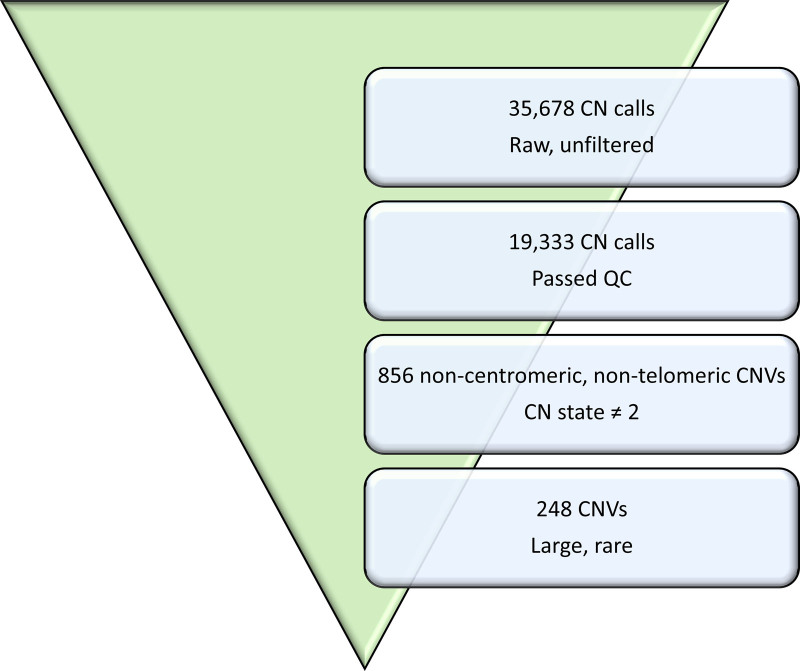
**Filtering of chromosomal copy number data of 89 nonsyndromic congenital heart disease cases.** CN indicates copy number; CNV, copy number variation; and QC, quality control.

All samples subjected to chromosomal microarray met the quality criteria. One TOF patient was found to have chromosomal aneuploidy (47, XXY) and was excluded from further CNV analysis. In the remaining 89 participants, 35 678 copy number calls were identified, which were filtered to 248 large, rare CNVs (Figure 1), comprising 89 microdeletions and 159 microduplications ranging in size from 100 kb to 6.1 Mb (10.25375/uct.11953749).

**Figure 2.**
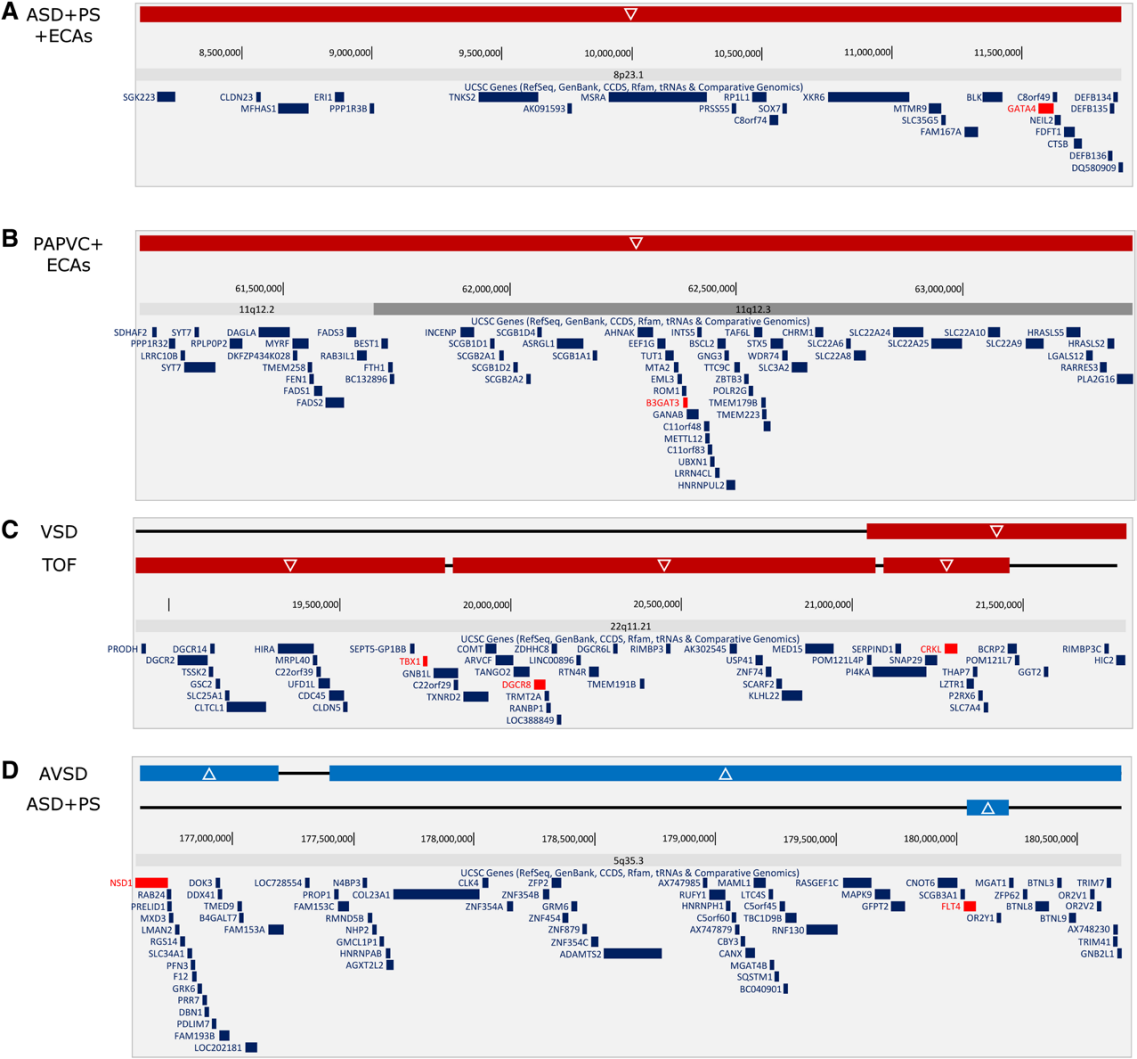
**Cytogenetic locations of the copy number variants identified to overlap with congenital heart disease genes in the study cohort. A**, Deleted region of 8p23.1 in a patient with atrial septal defect (ASD), pulmonary stenosis (PS), and extracardiac anomalies (ECAs; 2_00168). **B**, Deleted region of 11q12.3 in a patient with partial anomalous pulmonary venous connection (PAPVC) and ECAs (2_00018). **C**, Deletion regions of 22q11.21 in patients with ventricular septal defect (VSD) and Tetralogy of Fallot (TOF; 2_00162 and 2_00025). **D**, Duplicated regions of 5q35.3 in patients with atrioventricular septal defect (AVSD) and ASD with PS (2_00013 and 2_00097). Genes of interest are in red.

Of these, 13 CNVs of interest were identified (Table S1): eight encompassing known CHD genes (Table S2), and five encompassing candidate genes outside of the CHD gene list. The CNV set comprised five deletions and three duplications encompassing CHD genes (Table 3). Four of the identified CNVs, identified in three patients, were classified as pathogenic using American College of Medical Genetics and Genomics criteria. These included deletion of the 8p23.1 region encompassing the *GATA4* gene in a proband with atrial septal defect (ASD), pulmonary stenosis, and ECAs (Figure 2A); deletion of the 11q12.3 region encompassing the *B3GAT3* gene in a proband with partial anomalous pulmonary venous connection and ECAs (Figure 2B); and two deletions of the 22q11.2 region encompassing *TBX1* and *CRKL* in a proband with TOF who was previously unrecognized to have 22q11.2 deletion syndrome (Figure 2C). A third microdeletion was identified in the 22q11.2 region in this TOF patient, encompassing the candidate gene *DGCR8*.

**Table 3.**
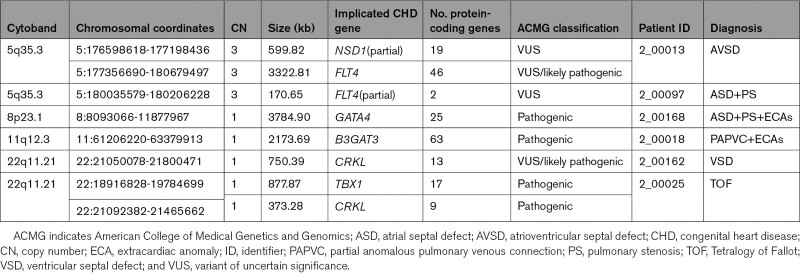
CNVs Overlapping With Known CHD Genes in the Study Cohort

Out of the four variants of uncertain significance identified, two were considered likely pathogenic due to reports of similar pathogenic or likely pathogenic CNVs in patients with CHD in the Database of Genomic Variation and Phenotype in Humans using Ensembl Resources (DECIPHER) and in the literature. These included a deletion on 22q11.2, which is smaller than the typically deleted region in 22q11.2 deletion syndrome, not overlapping *TBX1* but encompassing *CRKL*, which has been proposed as an independent causative gene for CHD in the 22q11.2 region,^13^ found in a patient with isolated ventricular septal defect (Figure 2C); and a duplication of two adjacent regions on chromosome 5 encompassing the genes *FLT4* and *NSD1* (partially) in a patient with isolated atrioventricular septal defect (Figure 2D), of which *FLT4* duplication has been reported in TOF.^6,7,14^

### Identification of Candidate CHD Genes

**Figure 3.**
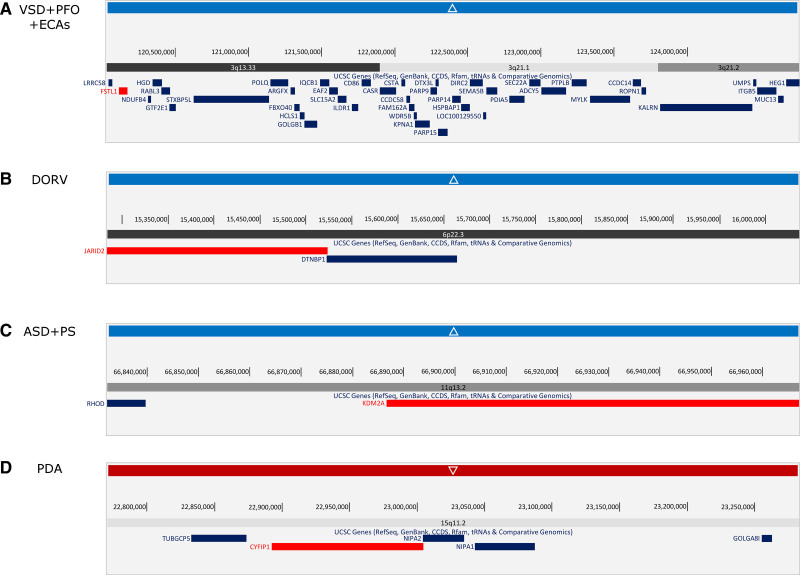
**Visualization of copy number variants overlapping candidate congenital heart disease genes. A**, Duplicated region of 3q13.33 – 3q21.2 in a patient with ventricular septal defect (VSD), patent foramen ovale (PFO), and extracardiac anomalies (ECAs; 2_00132). **B**, Duplicated region of 6p22.3 in a patient with DORV (2_00039). **C**, Duplicated region of 11q13.2 in a patient with atrial septal defect (ASD) and supravalval pulmonary stenosis (PS) (2_00036). **D**, Deleted region of 15q11.2 in a patient with PDA (2_00192). Genes of interest are in red.

Interrogation of the remaining large, rare CNVs led to identification of five genes that may be involved in the development of CHD, namely *DGCR8, FSTL1, JARID2*, *KDM2A*, and *CYFIP1* (Table 4). *DGCR8* occurred in the 22q11.2 microdeletion region, in the same TOF patient with *TBX1*- and *CRKL*-encompassing deletions (Figure 2C), while *CYFIP1* occurs in the 15q11.2 locus that has been putatively associated with various types of CHD (Figure 3A).^15^ In contrast, the dosage sensitivity of the other three genes has not been established or associated with CHD in humans (Figure 3B through 3D), and no patients with CHD or other disorders were found to harbor similar CNVs in DECIPHER. These genes were identified as CHD candidates due to their high probability of loss-of-function intolerance scores combined with their expression in the developing murine heart and the reported cardiac and embryonic phenotypes in mouse models.

**Table 4.**
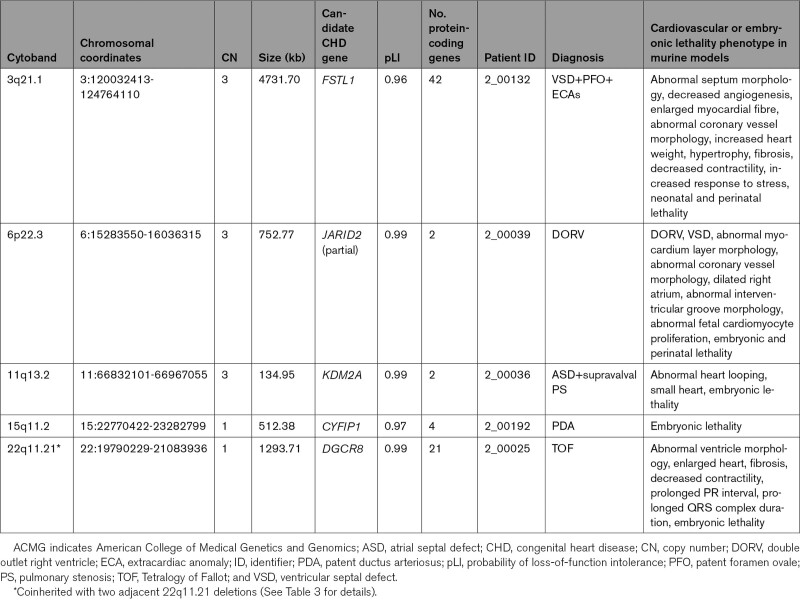
Candidate CHD Genes Identified in CNVs in the Cohort

## Discussion

In this study, we found likely causative CNVs covering known CHD-associated genes in 5/89 patients (5.6%), as well as a single patient with a previously unrecognized 47, XXY karyotype, yielding a total discovery rate of 6.7%. Our study shows that chromosomal microarray in South African patients yielded similar results to international studies, predominantly conducted in patients of European ancestry: the CNV detection rate of 5.6% is in accordance with the internationally reported range of 3% to 25%.^10,16^ These results highlight the wide genetic heterogeneity of CHD, as the identified CNVs overlapped six different genes that have been previously associated with CHD, as well as five additional candidate CHD genes, in ten unrelated probands with a variety of CHD phenotypes including ECAs such as autism spectrum disorder and dysmorphisms.

The interpretation of chromosomal structural changes is a challenge. Approximately 12% of the human genome consists of frequently occurring CNVs that have no phenotypic consequence.^17^ It is therefore important to distinguish truly causative microduplications and microdeletions from benign CNVs commonly found in the general population. The genotype-phenotype relationship is often complex and difficult to establish.^18^ CNV pathogenicity is typically determined based on CNV size, gene content, frequency in healthy individuals, and comparison with peer-reviewed publications and/or public databases.^19^ Accordingly, our approach to CNV data curation involved stringent filtering of CNVs, the use of a gene list comprising 153 CHD-associated genes, comparison to a control population totaling 475 841 healthy individuals, and comparison to patients with similar chromosomal imbalances and disease phenotypes in the literature. Using this approach, we were able to identify CNVs overlapping genes known to cause CHD, namely *GATA4, TBX1, CRKL, FLT4, NSD1*, and *B3GAT3.*

Associations of *GATA4* (8p23.1) deletion with ASD, pulmonary stenosis and ECAs; and of *TBX1* and *CRKL* (22q11.2) deletions with TOF and ventricular septal defect in this study are in accordance with previous reports.^4,10,16,18,20–25^ This includes the recent genomic analysis of 98 Nigerian CHD patients, in which a 3.8 Mb deletion encompassing *GATA4* and 26 other genes was described in a patient with various CHDs including pulmonary stenosis, mitral regurgitation, right ventricular hypoplasia, aortic hypoplasia, and a common atrium.^11^

*B3GAT3* encodes an enzyme that plays a critical role in the final step of proteoglycan biosynthesis.^26^ Family studies have reported that deleterious point mutations in the gene cause Larsen-like syndrome, a disorder characterized by short stature, multiple joint dislocations, craniofacial dysmorphic features as well as a wide spectrum of cardiac and/or aortic diseases including bicuspid aortic valve, mitral valve prolapse, ASD, ventricular septal defect, aortic root dilation, and pulmonary artery dilation.^27–29^ We identified a microdeletion of the 11q12.3 region including *B3GAT3* in a male proband with partial anomalous pulmonary venous connection and ECAs including dysmorphisms and developmental delay. While these phenotypic findings are in accordance with the *B3GAT3* associated-clinical phenotype described in the literature,^26–29^ this is to our knowledge the first description of a microdeletion in the region causing developmental and cardiovascular disease, although a 9.86 Mb duplication encompassing *B3GAT3* (and incidentally *KDM2A*, a candidate identified in this study) has been described in a DECIPHER participant (333571) with mitral valve prolapse, developmental delay, and other ECAs.

Two patients were found to have microduplications of the 5q35.5 region, one encompassing *FLT4* only and, in a second patient, a much larger duplication encompassing *FLT4* and also partially encompassing *NSD1*. Both *FLT4* duplications and loss-of-function mutations have been previously described in patients with TOF.^6,7,14,30^ This indicates that *FLT4* duplication may cause CHD phenotypes other than TOF, as the patients in our setting had atrioventricular septal defect and ASD with pulmonary stenosis. *NSD1* encodes a histone methyltransferase involved in the regulation of the epigenome during growth and development. While microdeletion and mutation of *NSD1* causes Sotos syndrome, duplications of this region, and specifically of *NSD1*, have been identified in individuals presenting with growth retardation, developmental delay, microcephaly, and CHDs such as ASD and aortic stenosis.^10,31–33^ In the patient with duplication of the longer region, it is not possible to ascribe the phenotype specifically to *NSD1* or *FLT4.* Our study thus identified CNVs in known regions likely to cause disease in five probands by American College of Medical Genetics and Genomics criteria.

We also investigated genes not conclusively associated with human CHD but with low tolerance of loss-of-function mutation in population databases, high mouse developmental cardiac expression, and CHD phenotypes demonstrated in the mouse, as potential candidates. The analysis identified five candidate genes, namely *DGCR8, JARID2, FSTL1, KDM2A*, and *CYFIP1.*

*DGCR8*, located within the 22q11.2 microdeletion syndrome region, encodes a subunit of the microprocessor complex involved in the biogenesis of microRNA, which plays an important role in global gene regulation.^34^ While the entire 22q11.2 cytoband is known to be associated with CHD, most research has focused on *TBX1* and *CRKL.*^10,18,23^ However, previous studies have suggested that there may be a whole set of genes in the region contributing to these phenotypes, including *DGCR8*.^34,35^ We were able to identify a microdeletion overlapping *DGCR8* in a proband diagnosed with TOF, and although this patient also had microdeletions encompassing *TBX1* and *CRKL*, which are likely the primary causative CNVs in this individual, this may indicate that the 22q11.2 locus is more complex than previously thought.

*CYFIP1* was identified as a candidate through documented embryonic lethality in mouse knockout studies.^36^ The gene occurs on 15q11.2, located between two breakpoints (BPs) for recurrent chromosome 15 CNVs, BP1 and BP2. This region is a hotspot for genetic disorders, with BP1-BP3 or BP2-BP3 microdeletions causing Prader-Willi syndrome or Angelman syndrome. The role of the smaller BP1-BP2 CNVs in disease is unclear at this stage but has been linked to neurological disorders such autism and intellectual disability^36^ and is a putative region for CHD, with 15q11.2 deletions described in isolated cardiac defects such as TOF and coarctation of the aorta,^15^ as well as syndromic CHDs occurring in conjunction with dysmorphisms and psychomotor phenotypes.^37^

In contrast, *JARID2*, *FSTL1*, and *KDM2A* occur in genomic regions that have not been conclusively associated with CHD or other disorders as yet, although these genes may be of interest due to their roles in cardiovascular development and the results of prior mouse models.^38–42^ Of note, *JARID2* has been shown to regulate *NOTCH1* expression^38^ and is itself regulated by the well-characterized CHD-associated gene *NKX2.5* during outflow tract morphogenesis.^39^ Two *JARID2* mutations have been described in the literature from exome sequencing studies, including a heterozygous loss-of-function mutation in aortic and pulmonary stenosis,^43^ and missense mutations in familial bicuspid aortic valve.^44^ No possibly causative variants in *FSTL1* or *KDM2A* have been reported, although *FSTL1* was recently screened for mutations in 54 patients with CHD and no variants found.^45^

Finally, the identification of a 47, XXY (Klinefelter syndrome) patient in this cohort is of interest. The role of this common (~1/600 males), yet under-diagnosed (25%–40%),^46^ form of aneuploidy in CHD is unclear. Analysis of 495 TOF patients found two with Klinefelter syndrome,^47^ and a smaller but more recent study demonstrated high incidence (5/44, 29.4%) of CHD among patients with Klinefelter syndrome.^48^ However, patients with congenital abnormalities are more likely to undergo genetic testing. It is currently thought that ascertainment bias may largely or entirely account for the apparent increased risk of CHD among Klinefelter syndrome males that some previous studies have reported.^49^ Large-scale population-based studies of Klinefelter syndrome that would accurately quantify any true increase in CHD risk are lacking. The patient was investigated separately for pathogenic CNVs, and none found (Table S3).

This study has limitations. First, it is based on a small sample of participants. However, findings were congruent with those in larger populations, chiefly of European ancestry, described to date. Second, due to a lack of parental genotype data, we were unable to determine whether the detected CNVs were de novo or inherited. Finally, an ethnically matched control group could not be included in the filtering process due to minimal genotyping of healthy individuals from the Cape mixed ancestry and Black African populations to date. To address this, our pilot study focused on CNVs overlapping well-characterized CHD genes, to improve confidence that the detected CNVs are true susceptibility variants, and used available CNV data from 3463 Tanzanian children for CNV frequency analysis.

While many CHD genetics studies have focused on patients of European descent, our study presents a CHD cohort comprised predominantly of individuals of Cape mixed ancestry (65.3%) and Black Africans (31.9%). The recruitment sites for this study were public hospitals in the Western Cape of South Africa, where Cape mixed ancestry is the predominant population group.^50^ The study cohort is therefore considered to be largely representative of the general ethnic composition of the Western Cape. The Cape mixed ancestry population is unique to South Africa and is one of the most admixed populations in the world.^50^ Therefore, studying and sequencing individuals from this unique population is advantageous, as this may help identify benign mutations in other populations, as well as provide genetic information that can be extrapolated to all world populations.

Our study led to the identification of likely causative CNVs in 5.6% of a South African cohort of probands diagnosed with various types of CHD. Furthermore, we identified five candidate genes that may play a role in the development of CHD and can be further investigated in future studies. Studying the genetics and environmental factors predisposing to CHD in African populations could help us to identify risk factors specific to the sub-Saharan African CHD population which could eventually translate to preventative measures to reduce the burden of CHD on the African continent.

## Article Information

### Acknowledgments

The authors acknowledge the work and contribution of all the medical and research staff associated with the PROTEA study and the Children’s Heart Disease Research Unit. This study makes use of data generated by the Database of genomic variation and phenotype in humans using Ensembl resources community. A full list of centers who contributed to the generation of the data is available from https://decipher.sanger.ac.uk/about/stats and via email from decipher@sanger.ac.uk. Funding for the DECIPHER project was provided by Wellcome.

### Sources of Funding

This work is based on the research supported, in part, by the National Research Foundation (NRF) of South Africa (Grant number 98563). Any finding, conclusion, or recommendation expressed in this material is that of the authors and the NRF does not accept any liability in this regard. N.A. Saacks has been funded by the NRF and the UK Medical Research Council (MRC). Dr Shaboodien has been funded by the NRF and Medical Research Council South Africa (MRC-SA). B.D. Keavney acknowledges support from the British Heart Foundation (CH/13/2/30154 and RG15/12/31616). Dr Zühlke has been funded by the MRC-SA, Research Capacity Development Mid-Career Scientist Programme, NRF, and through the African Research Leader award jointly by the UK MRC and the UK Department for International Development (DFID) under the MRC/DFID Concordat agreement. The PROTEA study was funded by the MRC (MR/P025463/1) as well as the Global Challenges Research Fund with the University of Manchester and the University of Cape Town.

### Disclosures

None.

### Supplemental Material

Supplemental Methods

Tables S1–S3

References^51^–^54^

## Supplementary Material
