# Supplementary material for: Investigation of Copy Number Variation in South African Patients With Congenital Heart Defects
Source: Circ Genom Precis Med. 2022 Oct 7;15(6):e003510. doi: 10.1161/CIRCGEN.121.003510 (PMC9770125; doi:10.1161/CIRCGEN.121.003510)
Supplement: Supplementary file 1 [file hcg-15-e003510-s001.pdf]

SUPPLEMENTAL MATERIAL

Supplemental methods

Data access

The data that support the findings of this study are available from the corresponding author upon reasonable request. The filtered CNV calls have been made publicly available (10.25375/uct.11953749). Data from consortia were accessed subject to the applicable data-sharing agreements.

Patient recruitment

CHD patients were prospectively recruited into the PROTEA clinical registry (PROTEA Aim 1) via convenience sampling by clinical members of the Children's Heart Disease Research Unit, University of Cape Town.[12] Recruitment was performed at Red Cross War Memorial Children's Hospital or Groote Schuur Hospital, Cape Town, South Africa. No population-based case finding was performed. To minimize selection bias, recruitment to the clinical registry was systematic and all patients referred to the Western Cape cardiology service were screened via folder review (for prevalent cases) and echocardiogram (for all incident and certain prevalent cases). All patients found to have structural CHD and fitting the inclusion and exclusion criteria were invited to participate in Aim 1. All patients with structural CHD as confirmed by echocardiogram were considered eligible for inclusion in PROTEA Aim 1, while those with isolated conduction or functional abnormalities, patent foramen ovale, peripheral pulmonary stenosis or patent ductus arteriosus in premature infants were excluded. Participants were selected from the clinical registry for genetic analysis (PROTEA Aim 2) via both purposive and convenience sampling. Participants scheduled for cardiac catheterization and cardiac surgery were preferentially selected, so that phlebotomy could be performed under anesthesia and any associated pain or distress minimized.

This PROTEA sub-study analysis consisted of 90 CHD patients for whom DNA was available at the time of investigation, and who met the inclusion and exclusion criteria above. In addition, patients with previously recognized specific genetic diagnoses or known chromosomal aberrations, that had been detected by standard techniques, were excluded. Patients were recruited at all ages and were eligible for the study if they presented with an isolated heart defect or CHD with additional extra-cardiac anomalies (ECAs). The Human Research Ethics Committee of the University of Cape Town approved all procedures (reference no. 389/2019). Written informed consent to participate in this study was obtained from all participants, or from

their legal guardians or next of kin where appropriate, as well as a peripheral blood sample for DNA extraction.

Genotyping and filtering

Genotyping was performed using the Affymetrix CytoScan HD platform (Affymetrix, Santa Clara, CA USA). CMA data that did not pass quality control criteria (mean absolute pairwise difference $\leq$ 0.25, SNP quality control $\geq$ 15.0, waviness-standard deviation $\leq$ 0.12) were excluded from further analysis. Germline CNVs were called using Affymetrix Power Tools software v2.11.0 and based on human genome build hg19. CNVs with > 70% overlap with centromeres, telomeres or regions of segmental duplication were excluded, as were CNVs of low quality, as assessed using Affymetrix Chromosome Analysis Suit (confidence < 0.85, marker count < 50, mean marker distance < 15 kb). CNVs > 100 kb in size were extracted from the data and assessed, as described below, for their frequencies and overlap with a CHD gene panel. Mosaic CNVs were not investigated in this study.

CNV control populations

Two published control datasets were used: UK Biobank healthy controls (n = 472,378),[51] and a healthy pediatric African population from Tanzania (n = 3,463).[52] The UK Biobank was used as described previously.[53] CNVs occurring in < 0.01 of either of the control populations were considered rare and analyzed further.

CHD gene list

Large, rare CNVs were then compared to a list of CHD-associated genes (Supplemental Table II). To identify CNVs occurring in gene regions known to be associated with CHD, all CNVs that overlapped with one or more CHD genes listed in the Genomics England PanelApp gene panel for non-syndromic CHD were investigated (available at https://panelapp.genomicsengland.co.uk/panels/212/). The Genomics England PanelApp is a publicly available crowdsourcing tool that represents a consensus of causative genes for many diseases. The genes in each panel are rated according to a traffic-light system, in which green represents genes with a high level of evidence for the gene-disease association, amber represents those with a moderate level of evidence, and red represents genes with minimal evidence. A list of the CHD genes in the panel alongside their genetic rating is presented in Supplemental Table II. These classifications are used to guide clinical interpretation, so all classifications (green, amber, red) were considered in this investigation to maximize discovery.

The PanelApp list was curated in-house with additional known and suspected genes for syndromic and non-syndromic CHD, that have been associated with CHD in at least one published paper. CNVs overlapping any of these genes, from PanelApp or the in-house list, were prioritized for further investigation and assessed using ACMG criteria for pathogenicity.[19]

Identification of candidate CHD genes

Once CNVs overlapping known CHD-associated genes were analyzed, candidate CHD genes were identified by filtering the remaining genes using ExAC probability of loss-of-function intolerance (pLI) ≥ 0.8.[54] To further refine this list, genes were considered candidates for CHD if expression in the embryonic mouse heart was reported in the Gene Expression Database (http://www.informatics.jax.org/expression), and if they had a recorded cardiac phenotype or caused embryonic lethality when mutated in mice, according to the Mouse Genome Informatics database (http://www.informatics.jax.org/).

Supplemental Tables

Supplemental Table I. CNVs of interest identified in the cohort by chromosomal microarray analysis

Patient ID	Chromosomal coordinates	Event	Size (kb)	Encompassed genes*
2_00013	5:176598618-176727216	Partial gene duplication	599.82	**NSD1**, RPL21P60, PRMT1P1
2_00013	5:177356690-180679497	Duplication	3,322.81	LOC100128340, SUDS3P1, PROP1, FAM153C, LOC100288656, RPL19P9, N4BP3, RMND5B, NHP2, LOC645853, GMCL1P1, HNRNPAB, PHYKPL, COL23A1, MRPL50P3, RN7SL646P, CLK4, RN7SKP70, VN2R2P, ZNF354A, AACSP1, LOC100129457, ZNF354B, RNU1-39P, ZFP2, LOC100422593, PIGFP1, ZNF454, GRM6, LOC100288803, ZNF879, ZNF354C, ADAMTS2, LOC100128622, LOC100289470, RUFY1, PRDX2P3, LOC101928445, HNRNPH1, C5orf60, LOC100502572, LOC105377763, CBY3, HMGB3P22, CANX, MAML1, LOC100884169, LTC4S, MGAT4B, MIR1229, SQSTM1, MRNIP, RN7SKP150, LOC100996419, TBC1D9B, RPS15AP18, RNF130, MIR340, LOC646058, RASGEF1C, RPS8P7, MAPK9, LOC100419721, GFPT2, RNU6-525P, CNOT6, LOC100329129, SCGB3A1, **FLT4**, LOC100420514, OR2AI1P, RNU1-17P, OR2Y1, MGAT1, HEIH, LINC00847, ZFP62, BTNL8, RPS29P12, LOC100128762, LOC100419853, LOC646227, BTNL3, RNU6-1036P, BTNL9, MIR8089, RPL13P10, TRNAV2, TRNAL34, FOXO1B, TRNAL24, TRNAV9, LOC729707, OR2V1, OR2V2, TRNAV5, TRL-AAG7-1, TRNAV3, TRNAV4, TRNAL4, TRNAL32, TRNAV10, TRNAP3,

				LINC01962, TRNAT1, TRIM7, LOC100507546, TRNAA26, TRNAK33, TRNAV1, TRNAK2, TRNAV6, MIR4638, TRIM41, RACK1, SNORD96A, LOC100289627, SNORD95, LOC100507619, CTC-338M12.4
2_00018	11:61206220-63379913	Deletion	2,173.69	SDHAF2, RN7SL23P, PPP1R32, MIR4488, LRRC10B, SYT7, LOC101927495, RPLP0P2, DAGLA, MYRF, DKFZP434K028, TMEM258, MIR611, FEN1, FADS1, MIR1908, FADS2, FADS3, MIR6746, RAB3IL1, RNU6-1243P, BEST1, FTH1, RPS2P37, LOC100507521, INCENP, EEF1DP8, SCGB1D1, SCGB2A1, SCGB1D2, SCGB2A2, SCGB1D4, NPM1P35, ASRGL1, RCC2P6, SCGB1A1, AHNAK, EEF1G, MIR6747, TUT1, MTA2, EML3, ROM1, **B3GAT3**, GANAB, INTS5, C11orf98, LBHD1, METTL12, SNORA57, UQCC3, UBXN1, LRRN4CL, BSCL2, HNRNPUL2-BSCL2, GNG3, HNRNPUL2, TTC9C, ZBTB3, POLR2G, TAF6L, TMEM179B, MIR6748, TMEM223, NXF1, MIR6514, STX5, RNU6-118P, LOC105369332, WDR74, RNU2-2P, SNHG1, SNORD22, SNORD31, SNORD30, SNORD29, SNORD28, SNORD27, SNORD26, SNORD25, SLC3A2, CHRM1, RN7SL259P, SLC22A6, SLC22A8, LOC100127954, LOC644436, SLC22A24, RPL29P22, SLC22A25, CCND2P1, SLC22A10, SLC22A9, HRASLS5, LOC100533643, LGALS12, TMSB4XP5, RARRES3, HRASLS2, PLA2G16
2_00025	22:18916828-19784699	Deletion	877.87	PRODH, DGCR5, LOC100287576, LOC100506454, DGCR9, DGCR10, DGCR2, DGCR11, LOC100129262, TSSK1A, DGCR14, TSSK2, GSC2, LINC01311, SLC25A1, CLTCL1, RPL34P35, DVL1P1, KRT18P62, HIRA, RN7SL168P, MRPL40, C22orf39,

				LOC100506503, UFD1L, CDC45, CLDN5, LINC00895, LOC100129254, LOC100420103, SEPT5, SEPT5-GP1BB, GP1BB, **TBX1**, GNB1L, RPL7AP70
2_00025	22:19790229-21083936	Deletion	1,293.71	GNB1L, C22orf29, TXNRD2, RPL8P5, COMT, MIR4761, ARVCF, TANGO2, MIR185, <u>DGCR8</u>, MIR3618, MIR1306, TRMT2A, MIR6816, RANBP1, ZDHHC8, CCDC188, LOC284865, LINC00896, RTN4R, MIR1286, LOC440792, DGCR6L, LOC729444, FAM230A, GGTLC3, TMEM191B, PI4KAP1, LOC100506613, RN7SKP131, RIMBP3, SUSD2P2, CA15P2, PPP1R26P2, GGTLC5P, LINC01660, PPP1R26P3, LOC101927859, USP41, ZNF74, RNU6-225P, SCARF2, KLHL22, LOC100420177, RNY1P9, RN7SL812P, KRT18P5, MED15, SMPD4P1, IGLL4P, LOC100421121, SLC9A3P2, ABHD17AP4, POM121L4P, BCRP5, TMEM191A, PI4KA
2_00025	22:21050078-21800471	Deletion	373.28	BCRP5, TMEM191A, PI4KA, SERPIND1, SNAP29, **CRKL**, LINC01637, AIFM3, LZTR1, THAP7, THAP7-AS1, TUBA3FP, P2RX6, SLC7A4, MIR649, P2RX6P, LRRC74B, TUBA3GP, BCRP2, POM121L7P, E2F6P2, LOC284861, GGT2, LOC100132705, E2F6P3, POM121L8P, BCRP6, LOC100996335, PPP1R26P5, SUSD2P1, RIMBP3B, RN7SKP63, HIC2
2_00036	11:66832101-66967055	Duplication	134.95	RHOD, <u>KDM2A</u>
2_00039	6:15283550-15522252	Partial gene duplication	752.77	<u>JARID2</u>, RNU6-522P, RNU6-645P
2_00097	5:180035579-180206228	Partial gene duplication	170.65	**FLT4**, LOC100420514, OR2AI1P, RNU1-17P, OR2Y1

2_00132	3:120032413-124764110	Duplication	4,731.70	LRRC58, FSTL1, MIR198, RPL34P9, NDUFB4, HGD, RABL3, MTCO1P29, MTCO2P29, GTF2E1, NAP1L1P3, LINC02049, STXBP5L, MIR5682, LOC100419755, LOC100422044, POLQ, RPL7AP11, ARGFX, FBXO40, HCLS1, RN7SL172P, RNU4-62P, GOLGB1, IQCB1, EAF2, SLC15A2, ILDR1, CD86, CASR, HNRNPA1P23, CSTA, CCDC58, FAM162A, WDR5B, LOC102723582, KPNA1, PARP9, DTX3L, PARP15, LOC100421636, EIF4BP8, PARP14, HSPBAP1, DIRC2, LINC02035, SEMA5B, PDIA5, MIR7110, SEC22A, ADCY5, HACD2, MYLK-AS1, MYLK, MYLK-AS2, CCDC14, ROPN1, KALRN, MIR5002, RPL7P15, MIR6083, RNU6-143P, LOC100420144, UMPS, MIR544B, RPS26P22, ITGB5, ENO1P3, MUC13, HEG1
2_00162	22:21092382-21465662	Deletion	750.39	PI4KA, SERPIND1, SNAP29, **CRKL**, LINC01637, AIFM3, LZTR1, THAP7, THAP7-AS1, TUBA3FP, P2RX6, SLC7A4, MIR649, P2RX6P, LRRC74B, TUBA3GP, BCRP2
2_00168	8:8093066-11877967	Deletion	3,784.90	FAM86B3P, ALG1L13P, PRAG1, RN7SL178P, CLDN23, MFHAS1, RNU6-682P, MRPS18CP2, RPL10P19, ERI1, MIR4660, PPP1R3B, LOC101929128, RNU6-1151P, LOC157273, RNU6-526P, LOC100420404, TNKS, MIR597, LINC00599, MIR124-1, MSRA, LOC100420053, LINCR-0001, PRSS52P, RNU6-729P, PRSS55, RP1L1, MIR4286, C8orf74, RNA5SP252, SOX7, PINX1, MIR1322, LOC101929229, XKR6, MIR598, LOC101929269, RPL17P29, RPL19P13, MTMR9, LOC100505734, SLC35G5, TDH, FAM167A-AS1, RN7SL293P, RNU6-1084P, FAM167A, BLK, LINC00208, **GATA4**, SNORA99, C8orf49, NEIL2, SUB1P1, FDFT1, CTSB, OR7E158P, OR7E161P, DEFB136, DEFB135, DEFB134, OR7E160P, LOC100421446

2_00192	15:22770422-23282799	Deletion	512.38	ELMO2P1, TUBGCP5, CYFIP1, NIPA2, NIPA1, LOC283683, LOC729900, LOC100132817, PDCD6IPP1, LOC100133165, LOC101269108, WHAMMP3, GOLGA8IP, RN7SL495P, HERC2P2

* Genes in bold are in the CHD gene list (Supplemental Table II), while genes that are underlined were identified as CHD candidate genes.

Supplemental Table II. List of CHD-associated genes used in filtering of CMA data

Gene symbol	Phenotype	Source
ABL1	CHD and skeletal malformations syndrome	PanelApp (green)
ACTC1	ASD	PanelApp (amber)
ACVR1	Fibrodysplasia ossificans progressiva	In-house gene list
ACVR2B	Heterotaxy	PanelApp (green)
ADAMTS10	Weill-Marchesani syndrome	In-house gene list
ALDH1A2	TOF	PanelApp (red)
ANKRD1	DCM, total anomalous pulmonary venous return	In-house gene list
ANKRD11	KBG syndrome	In-house gene list
ARID1A	Coffin-Siris syndrome	In-house gene list
ARID1B	Coffin-Siris syndrome	In-house gene list
ATP7A	Menkes disease	In-house gene list
B3GAT3	Multiple joint dislocations, short stature, craniofacial dysmorphism, with or without CHD	In-house gene list
BCOR	Syndromic microphthalmia	In-house gene list
BMPR2	Familial pulmonary hypertension	In-house gene list
BRAF	LEOPARD syndrome	In-house gene list
BVES	Limb-girdle muscular dystrophy	In-house gene list
CACNA1C	Long QT syndrome, Brugada syndrome	In-house gene list
CDK13	CHD, dysmorphic facial features and intellectual development disorder	In-house gene list
CDKL5	Encephalopathy	In-house gene list
CFAP53	Heterotaxy	PanelApp (green)
CFC1	Heterotaxy	PanelApp (green)
CHD4	Sifrim-Hitz-Weiss syndrome	In-house gene list
CHD7	CHARGE syndrome	In-house gene list
CHUK	Cocoon syndrome	In-house gene list
CITED2	ASD, VSD	PanelApp (red)
COX7B	Linear skin defects with multiple congenital anomalies	In-house gene list
CREBBP	Rubinstein-Taybi syndrome	In-house gene list
CRELD1	AVSD with heterotaxy	PanelApp (amber)
CRKL	TOF	PanelApp (red)
DDX11	Warsaw breakage syndrome	In-house gene list
DDX59	Orofaciodigital syndrome	In-house gene list

DISP1	TOF	PanelApp (red)
DLL4	Adams-Oliver syndrome	In-house gene list
DOCK6	Adams-Oliver syndrome	In-house gene list
EFTUD2	Mandibulofacial dysostosis	In-house gene list
EHMT1	Kleefstra syndrome	In-house gene list
ELN	Supravalvular aortic stenosis	PanelApp (green)
EVC	Ellis-van Creveld syndrome	In-house gene list
EVC2	Ellis-van Creveld syndrome	In-house gene list
EYA1	Branchiootic syndrome	In-house gene list
FANCL	Fanconi anemia	In-house gene list
FBN1	Marfan syndrome, MASS syndrome, geleophysic dysplasia	In-house gene list
FGFR2	Apert syndrome	In-house gene list
FLNA	Valvular dysplasia	PanelApp (green)
FLT4	TOF	PanelApp (red)
FOXA2	None	In-house gene list
FOXC1	Axenfeld-Rieger syndrome	In-house gene list
FOXC2	Lymphedema-distichiasis syndrome	In-house gene list
FOXH1	VSD, TGA	PanelApp (red)
FOXL1	HLHS	PanelApp (red)
GATA3	Hypoparathyroidism, sensorineural deafness, and renal dysplasia	In-house gene list
GATA4	ASD, AVSD, TOF, VSD	PanelApp (green)
GATA5	TOF	PanelApp (amber)
GATA6	TOF	PanelApp (green)
GDF1	TOF, DORV, TGA, heterotaxy	PanelApp (green)
GJA1	HLHS	PanelApp (red)
GJA5	Familial atrial fibrillation	In-house gene list
GPC3	Simpson-Golabi-Behmel syndrome	In-house gene list
GPC5	TOF	In-house gene list
HAND1	HLHS	In-house gene list
HAND2	TOF	PanelApp (red)
HEY2	AVSD, tricuspid atresia, aortic aneurysm	In-house gene list
HIBCH	3-hydroxyisobutryl-CoA hydrolase deficiency	In-house gene list
HOXA1	Bosley-Salih-Alorainy syndrome	In-house gene list

HRAS	Costello syndrome	In-house gene list
INVS	Infantine nephronophthisis	In-house gene list
IRX4	VSD	PanelApp (red)
JAG1	TOF, Alagille syndrome	PanelApp (green)
KANSL1	Koolen-De Vries syndrome	In-house gene list
KAT6A	Arboleda-Tham syndrome	In-house gene list
KAT6B	Genitopatellar syndrome, SBBYSS syndrome	In-house gene list
KCNH2	Long QT syndrome	In-house gene list
KDM6A	Kabuki syndrome	In-house gene list
KDR	Capillary infantile hemangioma	In-house gene list
KMT2A	Wiedemann-Steiner syndrome	In-house gene list
KMT2D	Kabuki syndrome	In-house gene list
KRAS	Noonan syndrome, craniofaciocutaneous syndrome	In-house gene list
LEFTY2	Heterotaxy	PanelApp (amber)
MAP2K1	Cardiofaciocutaneous syndrome	In-house gene list
MAP2K2	Cardiofaciocutaneous syndrome	In-house gene list
MED13L	TGA	PanelApp (red)
MEIS2	Cleft palate, cardiac defects and mental retardation	In-house gene list
MESP1	TOF, VSD	In-house gene list
MMP21	Heterotaxy	PanelApp (green)
MYBPC3	LVNC, HCM, DCM	In-house gene list
MYH11	Familial thoracic aortic aneurysm	In-house gene list
MYH6	ASD, Shone complex	PanelApp (green)
MYH7	LVNC, HCM, DCM	In-house gene list
MYOM2	TOF	PanelApp (red)
NFATC1	TOF	PanelApp (red)
NHS	Nance-Horan syndrome	In-house gene list
NIPBL	Cornelia de Lange syndrome	In-house gene list
NKX2-5	ASD, VSD, TOF, HLHS, atrioventricular conduction block, atrioventricular valve defects	PanelApp (green)
NKX2-6	TrA	PanelApp (red)
NODAL	Heterotaxy	PanelApp (green)
NOTCH1	Aortic valve disease	PanelApp (amber)
NOTCH2	Alagille syndrome	PanelApp (green)

NPHP3	Meckel syndrome, nephronophthisis, renal-hepatic-pancreatic dysplasia	In-house gene list
NPHP4	Nephronophthisis	In-house gene list
NR2F2	Multiple CHDs	PanelApp (green)
NRAS	Noonan syndrome	In-house gene list
NRP1	TOF	In-house gene list
NSD1	Sotos syndrome	In-house gene list
PEX7	Peroxisome biogenesis disorder	In-house gene list
PITX2	Axenfeld-Rieger syndrome	In-house gene list
PLXNA2	TOF	In-house gene list
PLXND1	TrA	PanelApp (red)
PQBP1	Renpenning syndrome	In-house gene list
PRDM6	PDA	In-house gene list
PRKD1	CHDs and ectodermal dysplasia	In-house gene list
PTPN11	Noonan syndrome, LEOPARD syndrome	In-house gene list
RAB23	Carpenter syndrome	In-house gene list
RAD21	Cornelia de Lange syndrome	In-house gene list
RAF1	Noonan syndrome, LEOPARD syndrome	In-house gene list
RAI1	Smith-Magenis syndrome	In-house gene list
RBM8A	Thrombocytopenia-absent radius syndrome	In-house gene list
RIT1	Noonan syndrome	In-house gene list
RPL11	Diamond-Blackfan anaemia	In-house gene list
RPSA	Congenital asplenia	PanelApp (red)
SALL1	Townes-Brocks syndrome	In-house gene list
SALL4	Duane-radial ray syndrome	In-house gene list
SEMA3D	TOF	PanelApp (red)
SEMA3E	CHARGE syndrome	In-house gene list
SF3B4	Acrofacial dysostosis	In-house gene list
SHOC2	Noonan-like syndrome	In-house gene list
SHROOM3	Heterotaxy	PanelApp (amber)
SMAD3	Loeys-Dietz syndrome	In-house gene list
SMAD4	Myhre syndrome	In-house gene list
SMAD6	Aortic valve disease	PanelApp (red)
SMARCA4	Coffin-Siris syndrome	In-house gene list
SMARCB1	Coffin-Siris syndrome	In-house gene list

SMC1A	Cornelia de Lange syndrome	In-house gene list
SMC3	Cornelia de Lange syndrome	In-house gene list
SNX8	TOF	In-house gene list
SON	ZTTK syndrome	In-house gene list
SOS1	Noonan syndrome	In-house gene list
STK11	Peutz-Jeghers syndrome	In-house gene list
STRA6	Syndromic microphthalmia	In-house gene list
TAB2	Non-syndromic CHDs	PanelApp (green)
TBX1	TOF	PanelApp (red)
TBX20	ASD	PanelApp (amber)
TBX5	Holt-Oram syndrome	PanelApp (green)
TDGF1	Forebrain defects	In-house gene list
TRAF7	Cardiac, facial, and digital anomalies with developmental delay	PanelApp (green)
TFAP2B	Char syndrome, PDA	In-house gene list
TGFBR1	Loeys-Dietz syndrome	In-house gene list
TLL1	ASD	PanelApp (red)
TMEM260	Structural heart defects and renal anomalies syndrome	In-house gene list
TTC37	Trichohepatoenteric syndrome	In-house gene list
UBR1	Johanson-Blizzard syndrome	In-house gene list
VEGFA	TOF	In-house gene list
ZFPM2	TOF	PanelApp (red)
ZIC3	Heterotaxy	PanelApp (green)

Abbreviations: ASD – atrial septal defect; AVSD – atrioventricular septal defect; CHD – congenital heart disease; DCM – dilated cardiomyopathy; DORV – double outlet right ventricle; HCM – hypertrophic cardiomyopathy; HLHS – hypoplastic left heart syndrome; LVNC – left ventricular noncompaction; PDA – patent ductus arteriosus; TGA – transposition of the great arteries; TOF – Tetralogy of Fallot; TrA – truncus arteriosus; VSD – ventricular septal defect

Supplemental Table III. Additional CNVs identified in the proband with Klinefelter syndrome

Chromosomal coordinates	CN	Size (kb)	Encompassed genes
1:849467-923023	3	73.56	LOC284600, LOC100130417, SAMD11, NOC2L, KLHL17, PLEKHN1, PERM1
2:48833179-48908728	3	75.55	STON1-GTF2A1L, GTF2A1L, TPT1P11
5:158840054-158997220	3	157.17	LINC01845
10:135357401-135427143	3	69.74	SYCE1, SPRNP1, OR6L1P
11:4436061-4474701	3	38.64	OR51R1P, OR52P2P, OR52K2
14:106270454-106849677	3	579.22	IGH, IGHD, IGHM, LOC100288273, MIR4539, MIR4507, MIR4538, MIR4537, IGHJ6, IGHJ3P, IGHJ5, IGHJ4, IGHJ3, IGHJ2P, IGHJ2, IGHJ1, IGHD7-27, IGHJ1P, IGHD1-26, IGHD6-25, IGHD5-24, IGHD4-23, IGHD3-22, IGHD2-21, IGHD1-20, IGHD6-19, IGHD5-18, IGHD4-17, IGHD3-16, IGHD2-15, IGHD1-14, IGHD6-13, IGHD5-12, IGHD4-11, IGHD3-10, IGHD3-9, IGHD2-8, IGHD1-7, IGHD6-6, IGHD5-5, IGHD4-4, IGHD3-3, IGHD2-2, FAM30A, IGHD1-1, IGHV6-1, IGHVII-1-1, ADAM6, RPS8P1, IGHV1-2, IGHVIII-2-1, IGHV1-3, IGHV4-4, IGHV2-5, IGHVIII-5-1, IGHVIII-5-2, IGHV3-6, IGHV3-7, IGHV1-8, IGHV3-9, IGHV2-10, IGHV3-11, IGHVIII-11-1, IGHV1-12, IGHV3-13, IGHVIII-13-1, IGHV1-14, IGHV3-15, IGHVII-15-1, IGHV3-16, IGHVIII-16-1, IGHV1-17, SLC20A1P2, IGHV1-18, IGHV3-19, IGHV3-20, IGHVII-20-1, IGHV3-21, IGHV3-22, IGHVII-22-1, IGHVIII-22-2, IGHV3-23, IGHV1-24, HOMER2P2, LINC00226, IGHV3-25, IGHVIII-25-1, IGHV2-26, IGHVIII-26-1, IGHVII-26-2, IGHV7-27,

			IGHV4-28, IGHVII-28-1, IGHV3-29, GOLGA4P2, IGHV3-30, IGHVII-30-1, IGHV3-30-2, IGHV4-30-2, IGHV4-31, IGHVII-30-21, IGHVII-31-1, IGHV3-32, GOLGA4P1, IGHV3-33, IGHVII-33-1, IGHV3-33-2, IGHV4-34, IGHV7-34-1, LOC100288568, IGHV3-35, IGHV3-36
15:43888262-43968546	1	80.29	CKMT1B, STRC, RNU6-554P, CATSPER2, RNU6-610P, PDIA3P2, PPIP5K1P1
16: 17360916-17427230	1	66.32	XYLT1
17: 44213434-44292742	3	79.31	KANSL1, KANSL1-AS1
19:15780652-15820326	4	39.68	CYP4F12
